# Fine Needle Aspiration Cytology of Cysticercosis—A Case Report

**DOI:** 10.1155/2012/854704

**Published:** 2012-07-18

**Authors:** S. Suchitha, K. Vani, R. Sunila, G. V. Manjunath

**Affiliations:** Department of Pathology, JSS Medical College, JSS University, Karnataka, Mysore 570022, India

## Abstract

Cysticercosis, caused by cysticercus cellulosae, the larval form of *Taenia solium*, is potentially a dangerous systemic disease with variable clinical manifestations. The disease most commonly involves subcutaneous and muscle tissues, followed by the eye and brain. Cysticercosis can be diagnosed by various radiologic means or by serology, both of which, however, are not definitive. Biopsy and histologic examination containing the cysticerci is the most definitive method of diagnosis. We report a case of cysticercosis in a 27-year-old male patient, who presented with a subcutaneous swelling in the back which was diagnosed on fine needle aspiration cytology (FNAC).

## 1. Introduction

Fine needle aspiration cytology (FNAC) is a well-recognized diagnostic procedure for the evaluation of inflammatory nodules caused by parasites. The diagnostic role of FNAC in cysticercosis was first emphasized by Kung et al. in 1989 [1]. Since then spectrum of cytological details of cysticercosis covering the entire range, from viable cysts through necrotic and calcified lesions has been described [2]. We report a case of cysticercosis diagnosed by FNAC emphasizing certain simple interpretive aspects that are of practical value, especially to cytopathologists who do not have much experience in this field.

## 2. Case Report

A 27-year-old male patient presented with a swelling on the right side of the back of two week duration. The mass was 4 × 4.5 cm in size, soft in consistency, and was in the subcutaneous plane. It was not associated with pain. The clinical diagnosis was that of a lipoma. FNAC was done using 22 G needle and 10 mL syringe. Aspiration yielded straw colored clear fluid. The smears were air dried as well as wet fixed in 95% ethyl alcohol and stained with May Grunwald Giemsa, haematoxylin, and eosin and Papinicolaou stains.

**Figure 1:**
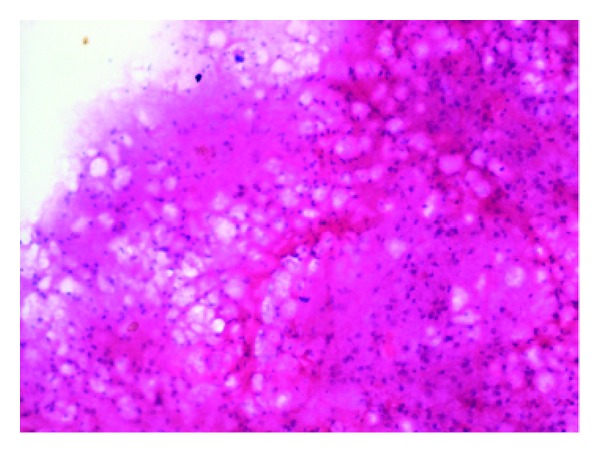
Multiple blue nuclei in a fibrillary background (H&E, ×400).

**Figure 2:**
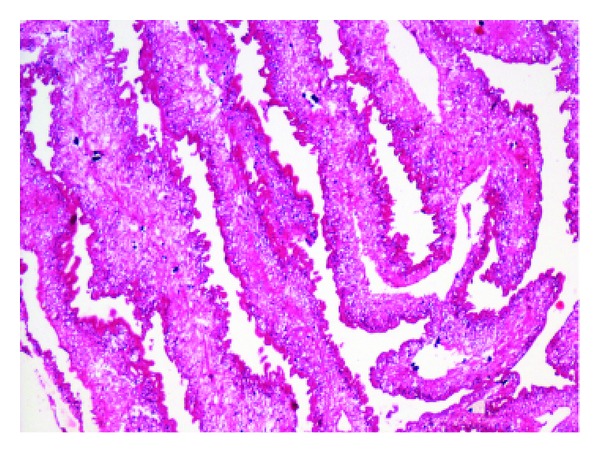
Cysticercus cellulosae (H&E, ×100).

On cytology, fragments of bluish fibrillary material with interspersed small nuclei were seen (Figure 1). Thick blue spherules within the substance of the fibrillary material gave a honeycomb appearance. Mixed inflammatory cells consisting of neutrophils, eosinophils, lymphocytes, and histiocytes were seen surrounding the fibrillary material. A diagnosis of parasitic material was offered on cytology. This material was excised and sent for histopathological examination. The parasite cysticercus cellulose was seen in the tissue section with extensive mixed inflammatory cell infiltrate in the surrounding tissue (Figure 2). A final diagnosis of subcutaneous cysticercosis was made. The patient also received oral antihelminthic therapy and is doing well.

## 3. Discussion

Human cysticercosis is a potentially dangerous systemic parasitic disease caused by the larval form of *T. solium*. It has a worldwide distribution including Central and Eastern Europe, South America, Africa, and tropical countries like India [3]. Humans acquire this infection by drinking contaminated water or consuming of undercooked pork that harbor the larva [4, 5]. Consumption of raw vegetables such as cabbage, radish, and carrots has also been known to cause the disease [5]. Though the parasite can be found in any organ, they are especially common in CNS, eyes, skeletal muscles, and subcutaneous tissues [6].

The palpable parasitic nodules are often clinically misinterpreted as benign mesenchymal tumors such as lipoma, neurofibroma, or as lymphadenopathy. Various diagnostic modalities employed to detect cysticercosis include radiology, serology, and pathological examination [6]. CT scan and MRI, though sensitive in diagnosing cysticercosis especially when the parasite involves the CNS, are very expensive. Moreover they provide only supportive diagnosis. Serological tests are useful if positive but cannot rule out the disease with negative results. False positivity is expected with the past parasitic infection or cross reactivity with other helminths. FNAC has emerged as a widely acceptable method for the diagnosis of cysticercosis [6].

Aspiration of clear fluid is a strong indicator towards parasitic infection in a palpable subcutaneous or intramuscular nodule [6]. The cytomorphology of cysticercosis varies from viable cysts through necrotic and calcified lesions. The most common finding in viable cysts are delicate fragments of bladder wall with tiny, parasitic nuclei in a clear, acellular background. Aspirates of necrotic lesions may contain fragments of bladder wall, invaginated portions, including calcerous corpuscles and detached single hooklets. The inflammatory background ranges from acute inflammation from prominent eosinophils, through granulomatous inflammation with necrosis, to acellular necrosis without significant residual inflammation. Single, detached hooklets and calcerous corpuscles may be the only recognizable remants in aspirates of calcified cysts [2]. In the present case aspiration of clear fluid and multiple nuclei in a blue fibrillary background with numerous inflammatory cells helped in arriving at a diagnosis of a parasitic cyst.

 Subcutaneous or intramuscular palpable parasitic nodule is most commonly due to cysticerci. Other parasites producing similar clinical presentation include coenuri, the larval form of tapeworm of the genus and Multiceps and spargana, larva of *Spirometra mansonoides* [6]. Hydatid cyst caused by the larva of* Echinococcus granulosus* can also occur in the subcutaneous tissue as an unusual presentation [2].

 Cytomorphological details of the aspirate help to differentiate each other. Cysticerci and coenuri have suckers and hooklets where as spargna do not. The Coenures has multiple protoscolices distinguishing from cysticerci which has a single scolex, which is difficult to see in cytological smears [6]. Bladder wall is thin and membranous in cysticerci whereas it is thicker and lamellated in a hydatid cyst. Multiple small scolices in the aspirate obtained from the hydatid cyst are in contrast to the single scolex of cysticerci [6]. However in the present case, only parenchymal layer of the larva having a loose fibrillary stroma with numerous interspersed nuclei was seen. No hooklets or scolex were seen in the smears. 

 Aspiration of clear fluid and varying proportions of inflammatory cells consisting of eosinophils, neutrophils, histiocytes, plasma cells, and giant cells in a palpable subcutaneous or intramuscular nodule should be viewed with high index of suspicion as that of parasitic origin even in the absence of identifiable parasitic fragments. The clear fluid aspirated from the nodules should be processed completely and should not be discarded as nonspecific. It is important to recognize the varied cytomorphology of cysticercosis. A diagnosis of cysticercosis is vital as it calls for a diligent search for the parasite in vital organs, where it causes significant morbidity and can even prove to be fatal.

## References

[1] Neelaiah S, Neha S, Qutubuddin C, Kalaivani A (2010). Fine needle aspiration cytology of cysticercosis. *Acta Cytologica*.

[2] Nanjeevan S, Vinod KA, Arati B (2006). Are all subcutaneous parasitic cysts cysticercosis?. *Acta Cytologica*.

[3] Neelam DK, Kiran M (1991). Fine-needle aspiration cytology of subcutaneous cysticercosis. *Diagnostic Cytopathology*.

[4] Gill M, Dua S, Gill P, Gupta V, Gupta S, Sen R (2010). Cytomorphological spectrum of subcutaneous and intramuscular cysticercosis: a study of 22 cases. *Journal of Cytology*.

[5] Rajwanshi A, Radhika S, Das A, Jayaram N, Banerjee CK (1991). Fine-needle aspiration cytology in the diagnosis of cysticercosis presenting as palpable nodules. *Diagnostic Cytopathology*.

[6] Handa U, Garg S, Mohan H (2008). Fine needle aspiration in the diagnosis of subcutaneous cysticercosis. *Diagnostic Cytopathology*.

